# MEP50/PRMT5-mediated methylation activates GLI1 in Hedgehog signalling through inhibition of ubiquitination by the ITCH/NUMB complex

**DOI:** 10.1038/s42003-018-0275-4

**Published:** 2019-01-18

**Authors:** Yoshinori Abe, Yosuke Suzuki, Kenji Kawamura, Nobuyuki Tanaka

**Affiliations:** Department of Molecular Oncology, Institute for Advanced Medical Sciences, Nippon Medical School, Kosugi-cho 1-396, Nakahara-ku, Kawasaki, Kanagawa 211-8533 Japan

## Abstract

Transcription factor GLI1 is an effecter of Hedgehog (HH) signalling and activated in a broad spectrum of cancers. However, the role of the HH-GLI1 pathway in cancer and the activation mechanism of GLI1 in HH signalling after dissociation from its inhibitor, SUFU, are not fully understood. Here, we found that GLI1 associated with the methylosome protein 50 (MEP50)/protein arginine methyltransferase 5 (PRMT5) complex and was methylated. Association of MEP50/PRMT5 with GLI1 was enhanced and expression of MEP50 and PRMT5 was activated by HH signals, suggesting their role in positive feedback regulation. Methylated GLI1 lost its ability to bind ubiquitin ligase ITCH/NUMB, resulting in nuclear accumulation and activation of GLI1. Moreover, protein expression of GLI1 was enhanced by MEP50/PRMT5 and expression of MEP50, PRMT5, and GLI1 target genes was upregulated in HH-expressing cancers. These results suggest that MEP50/PRMT5 is important for HH signal-induced GLI1 activation, especially in cancers.

## Abstract (web summary)

Yoshinori Abe et al. find that the Hedgehog (HH) signalling effector GLI1 is methylated by and associates with the MEP50/PRMT5 methyltransferase complex upon HH activation. They find that this complex plays a key role in positive feedback regulation of HH signalling in cancer.

## Introduction

G*LI1* was originally identified as an amplified gene in glioblastoma^1^, which functions as an effector of the HH signalling pathway^2,3^. The HH signalling pathway has central roles in the growth, patterning, and morphogenesis of many different regions within the body of vertebrates, insects, and most likely other invertebrates^4^. Moreover, it directs adult organ homoeostasis and stem cell maintenance^5–7^. In mammals, three related proteins, Sonic hedgehog (SHH), Desert Hedgehog (DHH), and Indian Hedgehog (IHH), function as ligands for their receptor, Patched1 (PTCH1), and binding of HH to PTCH1 alleviates PTCH1-mediated suppression of Smoothened (SMO), a member of the G protein-coupled receptor superfamily. Unsuppressed SMO subsequently enters primary cilia, small microtubule-based organelles, where it activates GLI family transcription factors^3,7^.

In mammalian cells, GLI family transcription factors include three members, GLI1, GLI2, and GLI3, all of which have five C_2_H_2_-Krüppel type zinc-finger motifs and are the only known transcriptional mediators of HH responses^3,7^. GLI1 contains only a C-terminal transcriptional activation domain, whereas both GLI2 and GLI3 possess C-terminal activation and N-terminal repression domains. In the absence of HH, GLI2, and GLI3 are phosphorylated at the base of cilia, resulting in proteolytic cleavage to generate their repressor forms (GLI2R and GLI3R). HH signalling changes the balance between the activator and repressor forms of GLI2 and GLI3 proteins by regulating their proteolytic cleavage, which increases the flux of GLI2, GLI3, and their inhibitor, suppressor of fused (SUFU), into cilia. In cilia, activated SMO inhibits SUFU to promote activation of GLI2 and GLI3, resulting in translocation of activated GLI2 and GLI3 from cilia to the nucleus^3,7^. In contrast, it has been shown that expression of GLI1 is low in unstimulated cells and induced by GLI2^8^, and that GLI1 also associates with SUFU and dissociates in response to HH signalling in cilia^9^. Therefore, it is considered that GLI1 functions as a positive feedback regulator and robust activator, which potentiates the transcriptional output of HH signalling.

GLI2R and GLI3R are generated from their full length proteins through limited proteasome-mediated protein degradation. In the absence of HH signals, GLI2 and GLI3 are sequentially phosphorylated by protein kinase A (PKA), casein kinase 1, and glycogen synthase kinase 3β^3,7^. These phosphorylations generate a binding site for F-box-containing protein β-transducin repeat-containing protein (βTrCP) that recruits the E3 ubiquitin ligase complex. Ubiquitinated GLI2 and GLI3 are targeted to the proteasome where the C-terminal transactivation domain is removed by partial degradation. In this process, GLI3 is efficiently processed to generate a repressor rather than GLI2^10^. Moreover, HH signalling-induced Speckle-type PDZ protein (SPOP) interacts with GLI2 and GLI3 and promotes their ubiquitin-mediated proteasomal degradation^11,12^. In contrast to GLI2 and GLI3, GLI1 is not a strong substrate of SPOP, but its protein levels are regulated by the adaptor protein NUMB that recruits GLI1 to the E3 ubiquitin ligase ITCH^13^.

Activation of HH signalling by overproduction of HH ligands, especially SHH and IHH, is widely observed in human cancers including those of the oesophagus, stomach, pancreas, and lungs^14–17^. It has also been shown that HH ligands expressed by cancer cells promote tumour growth indirectly by activation of HH signalling in the surrounding stroma, which creates a more favourable environment for tumour growth^18^. In the tumour microenvironment, it has been considered that HH signalling maintains the stemness of cancer stem cells^2,19^. GLI1 activation is also found in many cancers via both HH signalling-dependent and signalling-independent mechanisms^3^. Moreover, suppression of GLI1 expression in many types of cancer cells inhibits cell growth and invasiveness^20^, suggesting that GLI1 itself is important for cancer development. Indeed, both GLI1 and GLI2 activate a set of genes involved in the induction of cell growth, evading apoptosis, and epithelial-to-mesenchymal transition^3,7^. In this context, we previously demonstrated that GLI1 inhibits p53 protein accumulation through activation of ubiquitin-protein ligase MDM2^21^, suggesting that GLI1 overrides p53-mediated tumour suppression. However, the role of the HH-GLI1 pathway in cancer and the activation mechanism of GLI1 in HH signalling after dissociation from SUFU are not fully understood.

Here, we demonstrate that cytoplasmic GLI1 associates with methylosome protein 50 (MEP50), a component of the protein arginine methyltransferase complex, termed the methylosome, which contains protein arginine methyltransferase 5 (PRMT5)^22,23^, and assessed the role of the methylosome complex in regulation of HH signalling.

## Results

### MEP50 interacts with GLI1 via its WD repeats

**Fig. 1:**
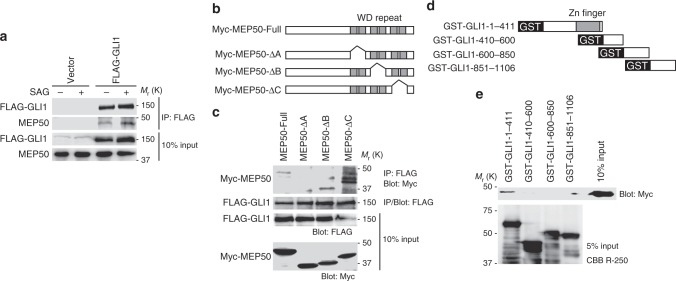
GLI1 interacts with the MEP50/PRMT5 complex. **a** FLAG-GLI1 interacted with endogenous MEP50 and interaction of FLAG-GLI1 and MEP50 was increased by HH signalling pathway activation. C3H10T1/2 cells were transfected with FLAG-GLI1 or the empty vector for 24 h and then treated with 300 nM SAG for an additional 24 h. Interaction of FLAG-GLI1 and MEP50 was detected by immunoprecipitation with anti-FLAG antibody followed by immunoblot analysis using anti-FLAG and anti-MEP50 antibodies. **b** Schematic structures of MEP50 deletion mutants. **c** Mapping of the GLI1-binding region in MEP50 by immunoprecipitation analysis. HEK293T cells were transfected with Myc-MEP50 deletion mutants and FLAG-GLI1 plasmids for 24 h. Interaction of FLAG-GLI1 and Myc-MEP50 deletion mutants was detected by immunoprecipitation with anti-FLAG antibody followed by immunoblot analysis using anti-FLAG and anti-Myc antibodies. **d** Schematic of GLI1 deletion mutants. **e** GST pull-down assays to map the MEP50-binding region in GLI1. GST-GLI1 deletion mutants coupled to glutathione sepharose were incubated with immunoprecipitated Myc-MEP50 from HEK293T cells. Immunoblotting was performed with an anti-Myc antibody. In **a** and **e**, data represent one of three independent experiments with similar results. In **c**, data represent one of two independent experiments with similar results. Unprocessed original scans of blots are shown in Supplementary Fig. 6

To understand the mechanism of GLI1 activation in HH signalling, we identified cytoplasmic GLI1-associated molecule(s) of HEK293 cells that stably expressed GLI1 with HA and FLAG tandem affinity tags. Tandem affinity purification using FLAG-agarose and HA-agarose beads followed by mass spectrometric analysis identified MEP50 (also called WD repeat domain 45 [WD45]) as a cytoplasmic GLI1-interacting protein (Supplementary Fig. 1a and b). MEP50 has also been identified as androgen receptor cofactor p44/WD repeat domain 77 (WDR77)^24,25^. The MEP50 protein contains 342 amino acid residues and six putative WD-40 repeats that function as scaffolds for protein–protein interactions^26,27^. To validate this interaction, we introduced expression vectors FLAG-tagged GLI1, Myc-tagged GLI2, or HA-tagged GLI3 into C3H10T1/2 cells and performed immunoprecipitation analyses to detect interactions between exogenous GLI family proteins and endogenous MEP50. As a result, MEP50 interacted with GLI1 (Fig. 1a), but not GLI2 or GLI3 (Supplementary Fig. 1c, d) in both the presence and absence of SAG, a SMO agonist that activates the HH pathway^28^. Furthermore, the GLI1–MEP50 interaction was enhanced by SAG (Fig. 1a).

We next determined the binding domains in both GLI1 and MEP50. Myc-tagged MEP50 and a series of WD-40 repeat deletion mutants were expressed in HEK293T cells that were subjected to immunoprecipitation analyses using an anti-Myc antibody. As shown in Fig. 1b, c, GLI1 interacted with the first two repeats of the six WD repeats in MEP50. Next, to determine the MEP50-binding domain of GLI1, a series of GST-GLI1-deleted proteins was expressed in *E. Coli*, and then purified and subjected to GST pull-down assays together with purified Myc-tagged MEP50 from HEK293T cells expressing Myc-MEP50. The GLI1 N-terminal region (1–411 aa), which contains the DNA-binding domain, bound to MEP50 (Fig. 1d, e). These results suggest that MEP50 directly and specifically interacts with GLI1.

### MEP50/PRMT5 methylosome induces GLI1 accumulation

**Fig. 2:**
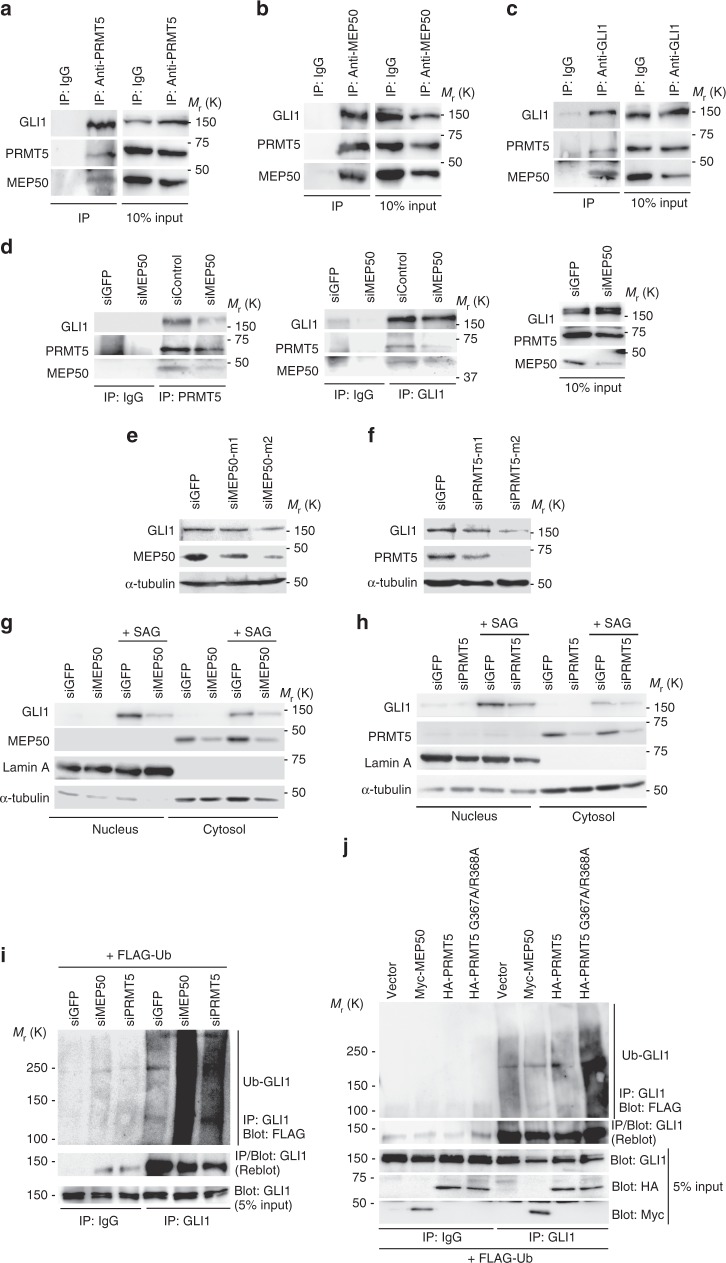
MEP50/PRMT5 complex supports GLI1 activation through GLI1 stabilisation downstream of the HH signalling pathway. **a–c** Endogenous GLI1/MEP50/PRMT5 complex in C3H10T1/2 cells. Cells were treated with SAG for 36 h, and complex was detected by immunoprecipitation (IP) with anti-PRMT5 (D5P2T) (**a**), anti-MEP50 (ERP10708 [B]) (**b**), or anti-GLI1 (V812) (**c**) antibodies, followed by immunoblot (IB) with antibodies against indicated proteins. **d** Dissociation of PRMT5 and GLI1 in stable MEP50 knockdown C3H10T1/2 cells by siMEP50-m2. Cells were treated with SAG for 48 h and treated with 50 μM MG132 for 4 h. GLI1/PRMT5 complex was detected by immunoprecipitation with anti-PRMT5 (D5P2T) or anti-GLI1 (V812) antibodies, followed by immunoblot with indicated antibodies. **e** Immunoblot of endogenous GLI1 in C3H10T1/2 cells expressing MEP50 siRNAs (siMEP50-m1 or siMEP50-m2). **f** Immunoblot of endogenous GLI1 in C3H10T1/2 cells expressing two independent PRMT5 siRNAs. **g** Immunoblot of nuclear and cytoplasmic GLI1 and MEP50 in stable MEP50-knockdown (siMEP50-m2) or control siGFP-expressing cells treated with 300 nM SAG. Cells were treated with SAG for 24 h and separated into cytosol and nucleus fractions. **h** Immunoblot analysis of endogenous nuclear and cytoplasmic GLI1 and PRMT5 in stable PRMT5-knockdown (siPRMT5-m2) C3H10T1/2 cells. Cells were treated with 300 nM SAG for 24 h and then separated as in **h**. **i** In vivo ubiquitination of GLI1 in C3H10T1/2 cells with or without expression of siMEP50. FLAG-ubiquitin was transfected into C3H10T1/2 cells. After 48 h of transfection, then cells were treated with 50 µM MG132 for 4 h. Endogenous ubiquitinated GLI1 was immunoprecipitated with an anti-GLI1 (C-1) antibody, followed by immunoblotting with indicated antibodies. **j** In vivo ubiquitination of GLI1 in C3H10T1/2 cells with exogenous expression of PRMT5 or MEP50. FLAG-ubiquitin and HA-PRMT5, HA-PRMT5 G367A/R368A (inactive form of PRMT5), or Myc-MEP50 were transfected into C3H10T1/2 cells. After 48 h, the cells were treated with 50 µM MG132 for 4 h. Endogenous ubiquitinated GLI1 was detected as described in **i**. In **a**, **i** and **j**, data represent one of two independent experiments with similar results. Unprocessed original scans of blots are shown in Supplementary Fig. 6

MEP50 is an adaptor molecule between PRMT5 and its substrates, such as cytoplasmic histone H2A and PIWI^29,30^. PRMT family proteins regulate the chromatin structure and expression of a wide spectrum of target genes, and PRMT5 regulates cell growth and survival pathways^31^. As shown in Fig. 2a, MEP50 and GLI1 were co-immunoprecipitated with PRMT5. These three proteins were also immunoprecipitated with anti-MEP50 or anti-GLI1 antibodies (Fig. 2b, c), suggesting that these proteins form a ternary complex in cells. Moreover, to avoid the effect of the reduced GLI1 protein level by MEP50 knockdown, we analysed the association of GLI1 with PRMT5 in the presence of proteasome inhibitor MG132. The binding of GLI1 to PRMT5 was attenuated by MEP50 knockdown even in the presence of proteasome inhibitor MG132, which inhibited the change in GLI1 protein expression (Fig. 2d), suggesting that the inhibition of this binding was not caused by reduced GLI1 protein due to MEP50 knockdown. In contrast, the GLI1/PRMT5 interaction was suppressed in siRNA-mediated MEP50 knockdown cells, suggesting that GLI1 interacts with PRMT5 via MEP50. Moreover, MEP50 knockdown suppressed GLI1 protein expression (Fig. 2e) that was restored by exogenous MEP50 expression (Supplementary Fig. 2a). Similar to MEP50, PRMT5 knockdown also inhibited GLI1 protein expression (Fig. 2f), suggesting that the MEP50/PRMT5 methylosome stabilises the GLI1 protein. Both MEP50 and PRMT5 were localised in the cytoplasm of C3H10T1/2 cells, and SAG enhanced GLI1 protein accumulation in both the cytoplasm and nucleus (Fig. 2g, h). Immunostaining of GLI1, MEP50, and PEMT5 proteins also revealed that these proteins were located in the cytoplasm, but that SAG stimulation induced protein accumulation in both the cytoplasm and nucleus of GLI1, but not MEP50 or PEMT5 (Supplementary Fig. 2b, c). Furthermore, cytoplasmic and nuclear accumulation of GLI1 by SAG was inhibited by MEP50 or PEMT5 knockdowns (Fig. 2g, h). These results suggest that HH signals induce GLI1 and MEP50/PRMT5 methylosome complex formation in the cytoplasm and GLI1 stabilisation, and that stabilised GLI1 accumulates in the nucleus. We also found that ubiquitination of GLI1 was enhanced by MEP50 or PRMT5 knockdowns (Fig. 2i). In addition, ectopic expression of Myc-tagged MEP50 or HA-tagged PRMT5 slightly suppressed the ubiquitination of GLI1 compared with empty vector-introduced cells (Fig. 2j). Moreover, the expression of enzymatically inactive PRMT5 (PRMT5 G367A/R368A)^32^, which functions in a dominant negative manner, induced marked enhancement of GLI1 ubiquitination (Fig. 2j). These results suggest that the MEP50/PRMT5 methylosome inhibits ubiquitination and proteasomal degradation of GLI1.

### HH signalling activates GLI1 activity via MEP50 and PRMT5

**Fig. 3:**
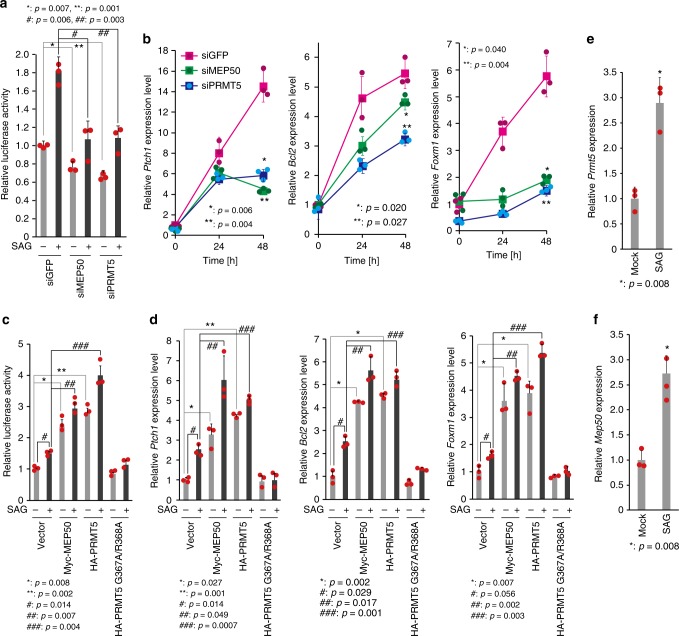
MEP50/PRMT5 complex-mediated GLI1 stabilisation enhances Gli transcriptional activity and HH signalling pathway activation induces *PRMT5* and *MEP50* expression. **a** Gli transcriptional activity in PRMT5 or MEP50 knockdown cells. siMEP50-m2 and siPRMT5-m2 siRNAs were stably expressed by recombinant retroviruses. A multimerized Gli-binding site luciferase reporter plasmid and phRL-TK control reporter plasmid were transfected into C3H10T1/2 cells. After 24 h of incubation, 300 nM SAG was applied for 24 h, and then luciferase assays were performed. **b** qRT-PCR analysis of *Ptch1*, *Bcl2*, and *Foxm1* expression in C3H10T1/2 cells with MEP50 knockdown or PRMT5 knockdown and treated with 300 nM SAG for the indicated times. siMEP50-m2 and siPRMT5-m2 siRNAs were stably expressed by recombinant retroviruses. **c** Gli transcriptional activity in HA-PRMT5 or Myc-MEP50-expressing cells. HA-PRMT5, HA-PRMT5 G367A/R368A, or Myc-MEP50 and a multimerized Gli-binding site luciferase reporter plasmid and phRL-TK control reporter plasmid were transfected into C3H10T1/2 cells. After 24 h of incubation, 300 nM SAG was applied for 24 h, and then luciferase assays were performed. **d** qRT-PCR analysis of *Ptch1*, *Bcl2*, and *Foxm1* expression in HA-PRMT5 or Myc-MEP50-expressing C3H10T1/2 cells. HA-PRMT5, HA-PRMT5 G367A/R368A, or Myc-MEP50 plasmids were transfected into C3H10T1/2 cells. After 24 h of incubation, cells were separated equally, and DMSO (−) or 300 nM SAG (+) were applied for 24 h. Protein levels are shown in Supplementary Fig. 3a. **e** and **f** qRT-PCR analysis of *PRMT5* (**e**) and *MEP50* (**f**) mRNA expression in C3H10T1/2 cells after 24 h of treatment with 300 nM SAG. In **a**–**c**, data represent one of two independent experiments with similar results. In **e** and **f**, data represent one of three independent experiments with similar results. The source data is shown in Supplementary Data 1

To determine the effects of MEP50 and PRMT5 on HH signalling, transcriptional activity of GLI1 was analysed following MEP50 or PRMT5 knockdowns in C3H10T1/2 cells. As shown in Fig. 3a, b, SAG-induced transcriptional activity of the GLI1-responsive luciferase reporter gene and mRNA expression of *Ptch1*, *Bcl2*, and *Foxm1*, major target genes of GLI1^33,34^, were suppressed by MEP50 or PRMT5 knockdowns. Moreover, SAG-induced transcriptional activity of GLI1 and expression of GLI1 target genes were slightly enhanced by ectopic expression of MEP50 or PRMT5, and markedly inhibited by dominant negative PRMT5 (PRMT5 G367A/R368A) (Fig. 3c, d and Supplementary Fig. 3a). The same effects of MEP50 and PRMT5 were also observed using the active form of SHH (SHH-N; Supplementary Fig. 3b, c). These results suggest that MEP50-mediated association of PRMT5 with GLI1-induced GLI1 protein accumulation, resulting in enhancement of GLI1 transcriptional activity. In addition, mRNA expression of *Prmt5* and *Mep50* was enhanced by SAG (Fig. 3e, f). The UCSC Genome Browser, a chromatin immunoprecipitation sequencing database, showed that overexpressed GLI2 and acetylated histone H3 at Lys27 (H3K27Ac), which distinguishes active enhancers from inactive enhancers^35^, were associated with the promoter region and exon 1 in *PRMT5* and *MEP50* genes (Supplementary Fig. 3d, e), suggesting that MEP50 and PRMT5 are positive feedback regulators in HH signalling.

### MEP50/PRMT5 methylosome induces GLI1 methylation

**Fig. 4:**
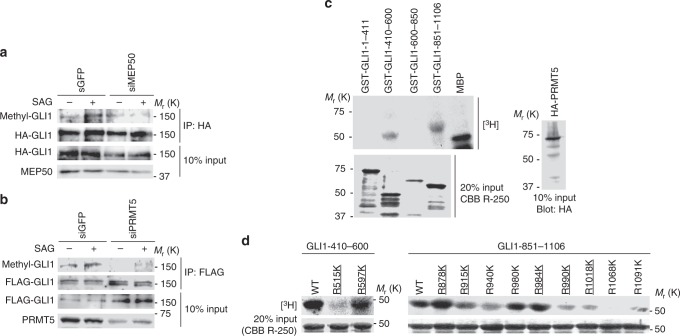
MEP50/PRMT5 complex induces GLI1 methylation. **a**, **b** Methylation of GLI1 in MEP50- (**a**) or PRMT5- (**b**) knockdown C3H10T1/2 cells. siMEP50-m2 and siPRMT5-m2 siRNAs were stably expressed by recombinant retroviruses. Cells transfected with FLAG-GLI1 were cultured for 24 h, followed by treatment with 300 nM SAG for 24 h. Methylated GLI1 was detected by immunoprecipitation with an anti-FLAG antibody followed by immunoblot with anti-SYM11 antibody. **c** In vitro methylation assays to determine the region including methylated arginine residues in GLI1 deletion mutants. HA-PRMT5 expression plasmid was transfected into HEK293T cells. At 48 h after transfection, the cells were lysed, and HA-PRMT5 was immunoprecipitated using an anti-HA (3F10) antibody. GST-GLI1 deletion mutants coupled to glutathione sepharose were incubated with immunoprecipitated HA-PRMT5 from HEK293T cells. Upper panel represents the methylated GST-GLI1 deletion mutant. Lower panel represents 20% input of GST-GLI1 deletion mutants detected by CBB R-250 staining. HA-PRMT5 expressed in 10% of total lysate used for immunoprecipitation is shown in the right panel. **d** In vitro methylation assays to determine methylation sites in GLI1 using amino acid substitutions (arginine to lysine) of candidate methylation sites. In vitro methylation assays were performed as described in (**c**). Upper panel represents methylated GST-GLI1 mutants. Lower panel represents 20% input of GST-GLI1 mutants detected by CBB R-250 staining. Underlined text denotes highly conserved residues among mammals, as shown in Supplementary Fig. 4. In **c**, data represent one of three independent experiments with similar results. In **a** and **d**, data represent one of twice independent experiments with similar results. Unprocessed original scans of blots are shown in Supplementary Fig. 6

As shown in Fig. 4a, b, SAG enhanced GLI1 methylation that was suppressed by MEP50 or PRMT5 knockdowns. In vitro methylation assays using a series of truncated GST-GLI1 fusion proteins demonstrated that two regions (410–600 and 851–1106) of GLI1 were methylated by PRMT5 (Fig. 4c). Two highly conserved arginine residues in the first region (410–600) and five arginine residues in the second region (851–1106) were determined as candidate methylation sites of GLI1 using the NEMO database (Supplementary Fig. 4). Amino acid substitutions (arginine to lysine) of the candidate methylation sites revealed that three arginine residues at 515, 990, and 1018 were methylated by PRMT5 (Fig. 4d). Although a previous study showed that PRMT1 methylates GLI1 at arginine 597, which promotes its transcriptional activity^36^, the amino acid at 597 was not targeted for MEP50/PRMT5-mediated GLI1 methylation (Fig. 4d).

### Methylated GLI1 inhibits association with ITCH/NUMB complex

**Fig. 5:**
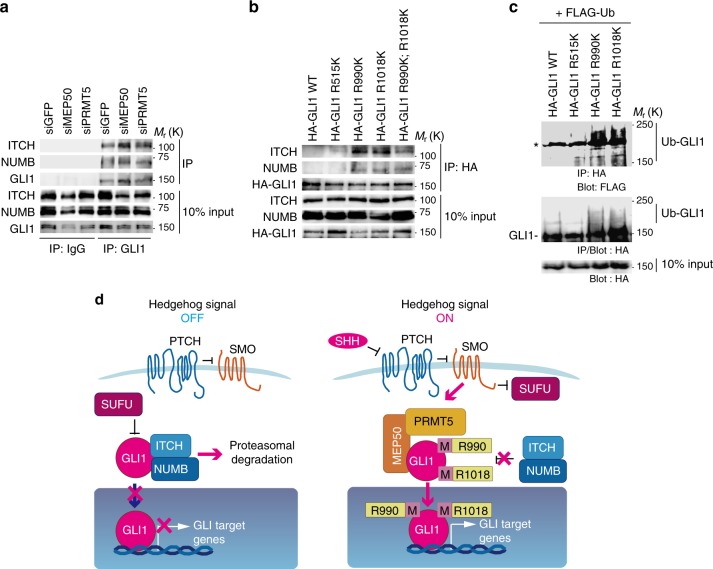
MEP50/PRMT5 complex-mediated GLI1 methylation inhibits the interaction of GLI1 with its E3 ligase complex, ITCH/NUMB, resulting in GLI1 stabilisation. **a** Interaction of GLI1 and endogenous ITCH or NUMB from stably PRMT5-knockdown or MEP50-knockdown C3H10T1/2 cells. siMEP50-m2 and siPRMT5-m2 siRNAs were stably expressed by recombinant retroviruses. MG132 (50 μM) was applied for 4 h before harvesting. **b** Interaction of GLI1 mutants with endogenous ITCH or NUMB in C3H10T1/2 cells. The cells were transfected as indicated. At 48 h post-transfection, 50 μM MG132 was applied for 4 h, and then the cells were lysed and subjected to immunoprecipitation with an anti-HA antibody, followed by immunoblotting with antibodies against the indicated proteins. **c** In vivo ubiquitination of HA-GLI1-RK mutants. Cells were transfected and cultured for 24 h, followed by treatment with 50 µM MG132 for 4 h before harvesting. Ubiquitinated GLI1 was detected by immuoprecipitation with an anti-HA (3F10) antibody and immunoblotting with anti-FLAG (upper panel) or anti-HA (lower panel) antibodies. The asterisk denotes non-specific bands. **d** Schematic diagram of the mechanism of PRMT5/MEP50-mediated GLI1 stabilisation. When the HH signalling pathway inactivates, the ITCH/NUMB E3 ligase complex binds to and ubiquitinates GLI1 for proteasomal degradation. In turn, under HH signalling pathway activation, the MEP50/PRMT5 complex methylates GLI1 to dissociate the ITCH/NUMB complex from GLI1, resulting in GLI1 stabilisation. Unprocessed original scans of blots are shown in Supplementary Fig. 6

It has been shown that ubiquitination of GLI1 is mediated by the ubiquitin ligase complex ITCH/NUMB^13^. We found that the GLI1 interaction with the ITCH/NUMB complex was increased in MEP50 or PRMT5 knockdown cells (Fig. 5a). Furthermore, substitution of arginine residues at 990 or 1018, but not 515, enhanced GLI1/ITCH/NUMB complex formation (Fig. 5b) and ubiquitination of GLI1 (Fig. 5c). However, we did not detect an additional effect of substitution at both 990 and 1018. These results suggest that MEP50/PRMT5-mediated GLI1 methylation stabilises GLI1 protein by inhibition of ITCH/NUMB-mediated GLI1 ubiquitination (Fig. 5d).

### MEP50/PRMT5-GLI1 axis operates in HH-expressing cancer cells

**Fig. 6:**
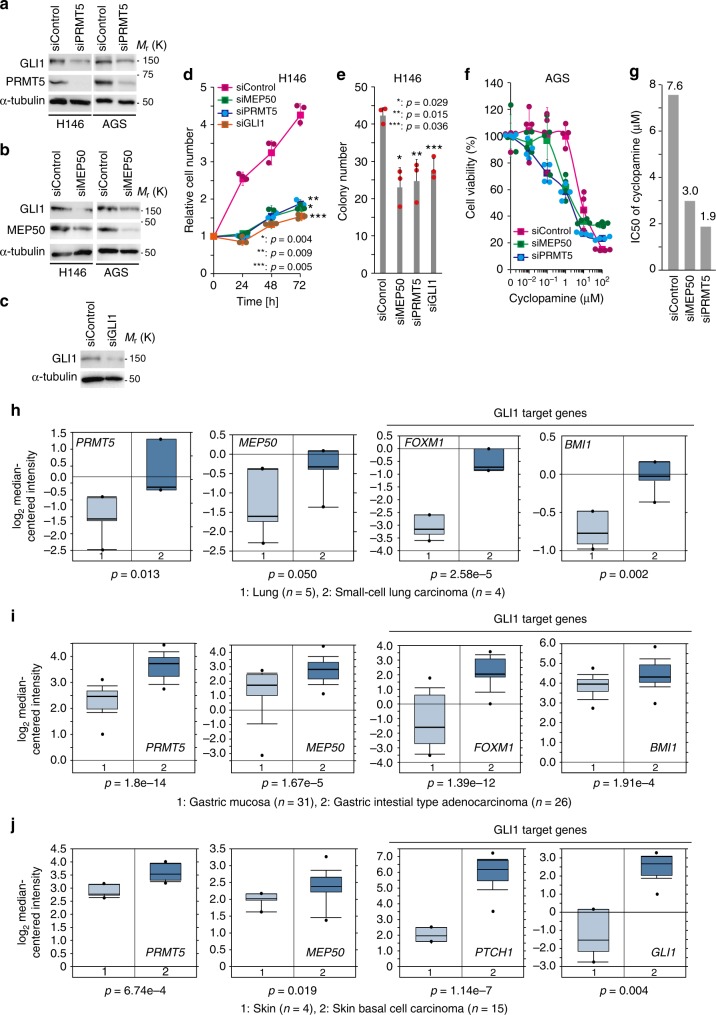
PRMT5 and MEP50 expression is upregulated in HH pathway-activated cancers, and PRMT5 inhibition is a potential therapeutic strategy for such cancers. **a**, **b** Immunoblot analysis of endogenous GLI1 in H146 and AGS cells stably expressing PRMT5 (**a**) or MEP50 (**b**) siRNAs. **c** Immunoblot analysis of endogenous GLI1 in H146 cells stably expressing GLI1 siRNA. In **a**–**c**, siRNAs were stably expressed via recombinant retroviruses. **d** Growth curves of PRMT5, MEP50, and GLI1-knockdown H146 SCLC cells. Results are shown in the mean ± s.d. of triplicate experiments. **e** A quantitative colony formation assay was performed by plating cells at a density of 1 × 10^4^ cells in a six-well plate and incubating them for 14 days. Surviving colonies were counted and represented as the mean ± s.d. of three independent wells. In **d** and **e**, siMEP50, siPRMT5, or siGLI1 was stably expressed via recombinant retrovirus in H146 cells. **f**, **g** IC_50_ values of cyclopamine in PRMT5-knockdown or MEP50-knockdown AGS cells. siRNAs were stably expressed via recombinant retroviruses. Cell viability (**e**) is shown as the mean ± s.d. *n* = 4. IC_50_ values of cyclopamine are shown in **g**. **h**–**j** Upregulated expression of *PRMT5*, *MEP50*, and GLI1 target genes in small cell lung carcinoma (**h**), gastric adenocarcinoma (**i**), and skin basal cell carcinoma (**j**) from the ONCOMINE database (https://www.oncomine.org/). The threshold of data was *p* ≤ 0.05. Each boxplot shows the log_2_ maximum, minimum, and median signal intensity of each mRNA from the corresponding expression array. Bold lines on each boxplot define the median value. *P*-values and sample numbers are indicated in each panel. Unprocessed original scans of blots are shown in Supplementary Fig. 6. Source data of **d**–**f** is shown in Supplementary Data 2

Activation of HH signalling by overproduction of SHH and IHH is widely observed in human cancers^14–17^. To understand the effects of MEP50/PRMT5 in HH-expressing cancer cells, we analysed the role of MEP50/PRMT5 in previously identified SHH-expressing gastric cancer cell line AGS^14^ and small cell lung cancer cell line H146^17^. As shown in Fig. 6a, b, protein expression of GLI1 was reduced by MEP50 or PRMT5 knockdowns, suggesting that MEP50/PRMT5-mediated activation of HH signalling operates in HH-expressing cancer cells. Moreover, similar to GLI1 knockdown in H146 cells, cell growth and colony formation were suppressed by MEP50 or PRMT5 knockdowns (Fig. 6c–e), suggesting that MEP50/PRMT5 are crucial for HH-induced oncogenesis. As described previously^14^, cyclopamine inhibited the growth of AGS cells (Fig. 6f), and 50% cell growth inhibition (IC_50_) was 7.6 μM (Fig. 6g). Moreover, MEP50 or PRMT5 knockdowns enhanced the growth inhibitory effect of cyclopamine (IC_50_: 3.0 and 1.9 μM, respectively). The expression of MEP50 and PRMT5 was still silenced after 72 h of cyclopamine treatment (Supplementary Fig. 5a). Moreover, the transcriptional activity of the GLI1-responsive luciferase reporter gene was suppressed by cyclopamine, and this suppression was also attenuated by MEP50 or PRMT5 knockdowns (Supplementary Fig. 5b). It is considerable that these effects may be caused by inhibition of residual GLI1 in knockdown cells (Fig. 6a) and inhibition of GLI2, which activates the same Gli-responsive element, through SMO inhibition by cyclopamine. Furthermore, these results suggest that inhibition of PRMT5 or methylosome complex formation may potentiate the inhibitory effect of cyclopamine against cancer cells.

Finally, examination of human cancer samples showed that expression of *PRMT5* and *MEP50*, as well as GLI1 target genes was upregulated in small cell lung carcinoma (Fig. 6h), gastric cancer (Fig. 6i and Supplementary Fig. 5c–e), other known HH signal-activated cancers including skin basal cell carcinoma (Fig. 6j)^3^ and breast cancer (Supplementary Fig. 5f–h).

## Discussion

Here, we identified the novel activation mechanism of GLI1 in HH signalling through MEP50/PRMT5-mediated methylation. Gene knockout studies in mice have shown that GLI2 and GLI3 are essential for embryogenesis. In contrast, GLI1 is dispensable for development, suggesting that GLI1 is not the primary mediator of HH signalling in embryogenesis^3^. A balance of activator GLI proteins (GLIA) and repressor GLI proteins (GLIR) regulates HH signalling, and GLI1 functions as a transcriptional activator. In contrast, GLI2/3 proteins are proteolytically cleaved to produce repressor forms, but HH signalling abrogates proteolytic cleavage, allowing full length active GLIs to induce target gene expression^3,7,37,38^. Therefore, unlike GLI2/3, although GLI1 is not essential for HH signalling in embryogenesis, it is possible that GLI1 functions as an enhancer of the HH signal intensity. For example, it has been shown that *Gli1* knockout or inhibition of HH signals in mice protects against development of obstructive injury-induced renal fibrosis as a failed wound-healing process provoked by chronic or repetitive injury^39^. Considering that HH is involved in cell proliferation in various tissues^4^, it is interesting that dysregulation of the HH-GLI1 pathway leads to cancer development. Therefore, elucidation of the activation mechanism of GLI1 by HH signals is also important to understand the mechanism of HH-GLI1-mediated cancer development.

Several lines of evidence show that GLI1 is regulated by protein modifications. For example, it has been shown that activation of PKA retains GLI1 in the cytoplasm through phosphorylation of GLI1 at a site near the nuclear localisation signal^40^. In the SHH-induced mouse model of medulloblastoma, highly expressed nonreceptor tyrosine kinase HCK phosphorylates GLI1, resulting in enhancement of GLI1-mediated target gene activation^41^. Moreover, IκB kinase β-mediated phosphorylation of GLI1 in response to tumour necrosis factor-α has been shown to increase GLI1 protein levels through inhibition of ITCH-mediated ubiquitination^42^. In addition to phosphorylation, acetylation of GLI1 causes inhibition of its transcriptional activity^43^. Furthermore, PRMT1, which belongs to the PRMT subfamily and is different from PRMT5, binds to GLI1 and methylates Arg597, resulting in enhancement of the transcriptional activity of GLI1 via an HH-independent pathway^29^. In contrast to this HH-independent activation system, MEP50/PRMT5 regulates HH-induced GLI1 protein accumulation in the nucleus and enhances HH signals by positive feedback activation through transcriptional induction. Therefore, we believe that this GLI1-activating mechanism is important to understand the fine regulatory mechanism of HH signalling. In the present study, the mechanism by which binding of GLI1 and MEP50/PRMT5 is enhanced by HH signals is still not fully elucidated. This is an interesting phenomenon that should be addressed further.

PRMT family proteins regulate many cellular processes, such as gene transcription, signal transduction, DNA repair, and mRNA splicing by arginine methylation, and enhancement of their methyltransferase activity is often associated with various cancers^44^. Knockdown of PRMT5 suppresses cell growth, and overexpression of PRMT5 causes hyperproliferation of cancer cells^31,45^. Moreover, increased enzymatic activity or expression of PRMT5 is observed in various human cancers, such as gastric, colorectal, and lung cancers, as well as leukaemia and lymphomas^44^. MEP50 is a component of the methylosome complex that contains PRMT5^22,23,46^. MEP50 expression has been associated with lung tumourigenesis and is crucial for lung cancer cell growth^47^. Previous studies have demonstrated involvement of the MEP50/PRMT5 methylosome in the proliferation of lung cancer cells^48^ and the invasive phenotypes of lung and breast carcinoma cancer cells^49^. However, to clarify the actual role of this activation pathway in tumourigenesis and cancer development requires further extensive studies of clinical cancer specimens that we are currently analysing. Our present results suggest that MEP50/PRMT5 might function in cancer cell proliferation and invasion at least in part via activation of GLI1.

Recently, SMO inhibitors have been developed and show utility in the treatment of basal cell carcinoma and the subtype of medulloblastoma dependent on HH signalling. Moreover, drugs targeting GLI1 have been developed, which are thought to have broader applicability^50^. In addition, arsenic trioxide (ATO) has been proposed to directly inhibit GLI1 proteins through binding to GLI1, resulting in inhibition of the transcriptional activity of GLI1^51^. Because ATO is an approved therapeutic^52^, there is the possibility of providing a therapy for cancer, in which HH signals are enhanced or *GLI1* gene expression is amplified. In addition to these treatments, considering that GLI1 is crucial for the development of cancer downstream of HH signalling, PRMT5 inhibitors^53^ and discovery of drugs targeting the binding of MEP50/PRMT5 and GLI1 may be useful for cancer treatment.

## Methods

### Cell culture

C3H10T1/2 cells were purchased from the Health Science Research Resources Bank. HEK293, HEK293T, AGS, and H146 cells were purchased from the American Type Culture Collection. C3H10T1/2 cells were maintained in Eagle’s basal medium (Sigma-Aldrich) supplemented with 10% foetal bovine serum (FBS). HA-FLAG-GLI1-expressing HEK293 cells were maintained in 293SFM medium (ThermoFisher Scientific) or Dulbecco’s modified Eagle’s medium (DMEM) supplemented with 10% FBS. HEK293 and HEK293T cells were maintained in DMEM supplemented with 10% FBS. AGS cells were maintained in DMEM/F-12 medium supplemented with 10% FBS. H146 cells were maintained in RPMI 1640 medium supplemented with 10% FBS.

### Antibodies and reagents

The following antibodies were used in analyses: anti-GLI1 (V812 [1:500 for immunoblotting, and 1:100 for immunoprecipitation], Cell Signalling Technology, 2534S; C-1 [1:200 for immunofluorescence staining, and 0.5 μg ml^−1^ for immunoprecipitation], Santa Cruz Biotechnology, sc-515751), anti-MEP50 (3F10 [1:1000], ABNOVA, H00079084-M01; EPR10708 (B) [1:100 for immunoprecipitation, 1:500 for western blotting, and 1:200 for immunofluorescence staining], Abcam), anti-PRMT5 (D5P2T [1:1000 for western blotting and 1:100 for immunoprecipitation] Cell Signalling Technology, 79998; EPR5772 [1:2000 for immunoblotting, and 1:50 for immunofluorescence staining], Abcam, ab109451), anti-lamin A/C (E-1 [1:500], Santa Cruz Biotechnology, sc-376248), anti-ITCH (EPR4936, 1:1000, Abcam, ab108515), anti-NUMB (1:2000, Abcam, ab14140), anti-symmetric methylated arginine (SYM11 [1:400], MERCK Millipore, 07-413), anti-FLAG (M2 [1:1000], Sigma, F1804), anti-Myc (9E10 [1:400], Santa Cruz Biotechnology, sc-40), anti-HA (3F10 [1:400], Roche Diagnostics, 11867423001; 16B12 [1:400], Covance, MMS-101R), anti-α-tubulin (DMIA [1:2000], Sigma-Aldrich, T9026), HRP-conjugated anti-rabbit IgG (1:10,000, GE Healthcare, NA9340-1ML), HRP-conjugated anti-mouse IgG (1:10,000, GE Healthcare, NA9310-1ML), HRP-conjugated anti-rat IgG (1:5000, Santa Cruz Biotechnology, sc-2006), Alexa-488-conjugated anti-rabbit IgG (1:400, Thermo Fisher Scientific, A-11070), and Alexa-568-conjugated anti-mouse IgG (1:400, Thermo Fisher Scientific, A-11019). SAG (SMO agonist) and cyclopamine were purchased from Merck Millipore, recombinant SHH-N was purchased from R&D Systems.

### Expression vectors

GLI1 cDNA with tandem HA and FLAG sequences was inserted into pBabe puro. GLI1 cDNA with the FLAG sequence was inserted into the pRK-5 vector. GLI1 and GLI3 cDNAs with the HA sequence were each inserted into the pEF vector. GLI2 cDNA with the Myc sequence was inserted into pcDNA3 vector, MEP50 cDNA with HA or Myc sequences was inserted into the pcDNA3 vector. PRMT5 cDNA with the HA sequence was inserted into pcDNA3. PCR primers for cloning of human MEP50 and PRMT5 cDNA are shown in Table 1. PRMT5 G367A/R368A mutant was generated by PCR using the pcDNA3-HA-PRMT5 expression plasmid as a template. HA-GLI1 amino acid substitutions were generated by PCR using the pEF-HA-GLI1 expression plasmid as a template. Myc-tagged MEP50-∆A–C mutants were generated by PCR using the pcDNA3-Myc-MEP50 expression plasmid as a template. To generate GST-tagged GLI1 deletion proteins, GLI1 cDNA fragments were subcloned by PCR and inserted into the pGEX-6P-1 vector (GE Healthcare). GST-GLI1 amino acid substitutions were generated by PCR using pGEX6P-1-GST-GLI1 deletion mutant plasmids as a template. Inserted cDNAs were checked by universal primers included in each vectors.

**Table 1:** Primer sequences used for PRMT5 and MEP50 cloning

Gene	Primer sequence
MEP50 (NM_001317062.1)	Forward: cggaattcATGCGGAAGGAAACCCCACCC
	Reverse: gctctagaCTACTCAGTAACACTTGCAGG
PRMT5 (NM_001039619.1)	Forward: cggaattcATGCGGGGTCCGAACTGGGGG
	Reverse: cggaattcCTAGAGGCCAATGGTATATGA

cDNA pool derived from HeLa cells was used for MEP50 and PRMT5 cloning

### Cell transfection and infection

Cells were passaged at 1 day prior to transfection and cultured to 50% confluence overnight in antibiotic-free medium containing 10% FBS. Cells were transfected with expression plasmids using GeneJuice (Merck Millipore), according to the manufacturer’s instructions. Retroviral infection was performed as described previously^54^. Briefly, equal amount of pBabe puro or pSUPER retro puro and helper DNA (psi2 for mouse cells and pPAM3 for human cells) plasmids were transfected into HEK293T cells. Twenty hours after transfection, medium was changed, and cells were further incubated for 24 h. Then, medium containing recombinant retrovirus was collected and removed debris using syrinde filter (pore size 0.45 μm; Corning) and used for infection to cells. Infected cells were selected by puromycin (Sigma-Aldrich: 1 μg ml^−1^).

### Tandem affinity purification of GLI1 complexes

The epitope-tagging strategy to isolate GLI1-containing protein complexes from human cells was performed essentially as described previously^55^. HEK293 cells infected with HA-FLAG-Gli1 recombinant retrovirus were grown in 293SFM medium and harvested at near confluency (~1 × 10^9^ cells). The cells were separated into cytoplasmic and nuclear fractions. Cytoplasmic fraction was dialysed for 5 h in a buffer consisting of 20 mM Hepes–KOH (pH 7.9), 20% glycerol, 0.1 M KCl, 0.2 mM EDTA, 0.5 mM PMSF, and 0.5 mM DTT. The lysate was centrifuged at 15,000×*g* for 15 min at 4 °C. The supernatants were immunoprecipitated with anti-FLAG antibody-conjugated M2 agarose (400 μl; Sigma-Aldrich). Bound proteins eluted 0.4 mg ml^−1^ FLAG peptide (Sigma-Aldrich) were further affinity purified using 20 μl anti-HA antibody-conjugated agarose (Roche Diagnostics). The final elutes from HA beads eluted with 2.5 mg ml^−1^ HA peptide (Roche Diagnostics) were separated by SDS–PAGE on a 5–20% gradient gel for silver staining analysis. Specific bands were excised from the gel and subjected to peptide sequencing by mass spectrometry using a 4700 proteomics analyser (AB SciEx). Data were analysed by the Mascot search engine (Matrix Science).

### GST fusion protein purification

GST-GLI1 fusion proteins were expressed in *E. coli* Rosetta2 (Merck Millipore) or Codon Plus (Agilent Technology). *E. coli* were lysed in B-PER Protein Extraction Reagents (Thermo Fisher Scientific), according to the manufacturer’s instructions. Protein extracts were centrifuged at 15,000×*g* for 15 min at 4 °C, and the supernatant (5 µg protein) was incubated with 20 µl glutathione sepharose 4B beads (GE Healthcare) for 2 h at 4 °C. The beads were then washed three times with PBS or methylation reaction buffer (MRE; 50 mM Tris–HCl [pH 7.5], 0.1 mM EDTA, and 50 mM NaCl).

### Immunoprecipitation and immunoblotting

Transfected or infected cells were lysed in TNE lysis buffer (1% Nonidet P-40, 10 mM Tris–HCl [pH 8.0], 150 mM NaCl, 1 mM EDTA, 1 mM NaF, 1 mM orthovanadate, 0.1 mM DTT, and a protease inhibitor cocktail [Nacalai Tesque]). Protein concentrations were quantified by a protein assay kit (Bio-Rad). Equal amounts of protein were used for immunoblotting and immunoprecipitation. For immunoprecipitation, lysates were incubated with the indicated antibodies and protein-A/G sepharose beads or agarose–antibody conjugates for 3 h at 4 °C. The beads were collected and washed three times in TNE buffer before denaturation and SDS–PAGE, followed by immunoblotting. Proteins were transferred to nitrocellulose membranes and probed with the indicated antibodies. Chemi-Lumi ONE Ultra (Nacalai Tesque) and a LAS-3000 mini Lumino-image analyser (GE Healthcare) were used to visualise antibody binding. To detect the ubiquitinated form of GLI1 in cells, 1 × 10^6^ C3H10T1/2 cells were transfected with 5 µg flag-tagged ubiquitin. At 48 h after transfection, the cells were treated with 50 µM MG132 (Calbiochem) for 4 h and then lysed in 25 µM MG132-containing TNE lysis buffer. Cell extracts were immunoprecipitated with an anti-GLI1 antibody (C-1, Santa Cruz Biotechnology) and analysed by immunoblotting. After detection of ubiquitinated GLI1, to detect immunoprecipitated GLI1, the anti-GLI1 antibody was stripped and re-blotting was performed with the anti-GLI1 antibody.

### Immunofluorescence staining

For immunofluorescence staining, 3 × 10^4^ C3H10T1/2 cells were seeded on a glass coverslip in a 12-well plate. The next day, 300 nM SAG was applied for 24 h at 37 °C. At 48 h after SAG treatment, the cells were fixed with 4% paraformaldehyde for 15 min at 37 °C and then permeabilized with 0.2% Triton X-100 for 7 min at room temperature. Fixed cells were incubated in 1% BSA-PBS for 30 min, followed by primary antibodies for 30 min at room temperature. After washing with PBS, the cells were incubated for 30 min at room temperature with Alexa Fluor 488-conjugated or Alexa Fluor 568-conjugated anti-rabbit or anti-mouse IgGs (Thermo Fisher Scientific). Finally, the cells were counterstained with Hoechst 33342 (Thermo Fisher Scientific) to visualise nuclei. Immunofluorescence was recorded using a FLUOVIEW FV1200 biological laser scanning microscope (Olympus). Images were captured using FLUOVIEW software (ver. 3.1-1, Olympus).

### RNAi

All shRNAs were inserted into the pSUPER retro puro vector (OligoEngine). The shRNA design was according to manufacturer’s instruction. The target sequences are listed in Table 2.

**Table 2:** shRNA sequences for RNAi experiments

siRNA	Sequence
siGFP	Sense: gatccccGGAGTTGTCCCAATTCTTGttcaagagaCAAGAATTGGGACAACTCCtttttggaa
	Anti-sense: agcttttccaaaaaGGAGTTGTCCCAATTCTTGtctcttgaaCAAGAATTGGGACAACTCCggg
Mouse siMEP50-m1	Sense: gatccccGCAAGGACTTGCATTGTTAttcaagagaTAACAATGCAAGTCCTTGCttttta
	Anti-sense: agcttaaaaaGCAAGGACTTGCATTGTTAtctcttgaaTAACAATGCAAGTCCTTGCggg
Mouse siMEP50-m2	Sense: gatccccGCATGCATGGAACGTCAATttcaagagaATTGACGTTCCATGCATGCttttta
	Anti-sense: agcttaaaaaGCATGCATGGAACGTCAATtctcttgaaATTGACGTTCCATGCATGCggg
Mouse siPRMT5-m1	Sense: gatccccGACTCTAGTATCAAGGAAttcaagagaTTCCTTGATACTAGAGTCCttttta
	Anti-sense: agcttaaaaaGACTCTAGTATCAAGGAtctcttgaaTTCCTTGATACTAGAGTCCggg
Mouse siPRMT5-m2	Sense: gatccccGGAAACAAATAAAGTGAAGttcaagagaCTTCACTTTATTTGTTTCCttttta
	Anti-sense: agcttaaaaaGGAAACAAATAAAGTGAAGtctcttgaaCTTCACTTTATTTGTTTCCggg
Human siControl	Sense: gatccccATCTCGCTTGGGCGAGAGTAAGttcaagagaCTTACTCTCGCCCAAGCGAGATttttta
	Anti-sense: agcttaaaaaATCTCGCTTGGGCGAGAGTAAGtctcttgaaCTTACTCTCGCCCAAGCGAGATggg
Human siMEP50	Sense: gatccccGGACTCTGTGTTTCTTTCAttcaagagaTGAAAGAAACACAGAGTCCttttta
	Anti-sense: agcttaaaaaGGACTCTGTGTTTCTTTCAtctcttgaaTGAAAGAAACACAGAGTCCggg
Human siPRMT5	Sense: gatccccGGAATCTCAGACATATGAAGTttcaagagaACTTCATATGTCTGAGATTCCttttta
	Anti-sense: agcttaaaaaGGAATCTCAGACATATGAAGTtctcttgaaACTTCATATGTCTGAGATTCCggg
Human siGLI1	Sense: gatccccGCAGTAAAGCCTTCAGCAAttcaagagaTTGCTGAAGGCTTTACTGCttttta
	Anti-sense: agcttaaaaaGCAGTAAAGCCTTCAGCAAtctcttgaaTTGCTGAAGGCTTTACTGCggg

siGFP and non-targeting control siRNA (siControl) were used as controls in RNAi experiments

### Luciferase assay

Firefly and Renilla luciferase activities were assayed with a dual luciferase assay system (Promega), according to the manufacturer’s instructions. Results are expressed as Firefly/Renilla ratios from triplicated experiments and represented as the mean ± s.d.

### RNA isolation and qRT-PCR

RNA isolation was performed using the NucleoSpin RNA II kit (Takara Bio). Equal amounts of RNA (0.5 µg) were reverse transcribed by a PrimeScript^®^ II 1st strand cDNA Synthesis Kit (Takara Bio). The resulting cDNA was subjected to qRT-PCR analysis using TaqMan Gene Expression Assay Mastermix and qRT-PCR probes (Thermo Fisher Scientific) with a StepOnePlus Real-Time PCR System (Thermo Fisher Scientific). The TaqMan gene expression assays used for mouse samples were *Ptch1* (Mm01306905_m1), *Gli1* (Mm00494645_m1), *Foxm1* (Mm00514924_m1), *Bcl2* (Mm00477631_m1), *Mep50* (Mm01296589_g1), and *Prmt5* (Mm00550472_m1). Each amplification reaction was performed in triplicate, and the mean and s.d. of three threshold cycles were used to calculate the amount of transcript in the sample (StepOne software v2.2.2; ThermoFisher Scientific). Quantification of mRNA was expressed in arbitrary units as the ratio of the sample quantity to the calibrator or to the mean values of control samples. All values were normalised to an endogenous control, mouse β-actin (Mm00607939_m1).

### In vitro methylation assay

HEK293T cells were transfected with HA-PRMT5 expression plasmids and immunoprecipitated with an anti-HA antibody. A recombinant substrate (5 μg) either GST-GLI1 protein or myelin basic protein (Sigma-Aldrich) as a positive control for a PRMT5 substrate, was incubated with immunoprecipitated HA-PRMT5. *S*-adenosyl-l-methyl-[^3^H] methionine (55 Ci mmol^−1^; Perkin Elmer) was added to each reaction in a total volume of 40 μl MRE, and samples were incubated for 1 h at 30 °C. Samples were separated by SDS–PAGE, and the gel was washed and incubated in amplify solution (Perkin Elmer). The gel was then dried and exposed on BIO-MAX MR X-ray film (Carestream) for 3–7 days at −80 °C.

### IC_50_ evaluation of cyclopamine

Cells (5 × 10^3^) were seeded in 96-well plates and cultured overnight. The cells were then exposed to increasing concentrations of cyclopamine ranging from 10 nM to 100 µM for 72 h. Cell viability was assessed by a CCK-8 staining kit (Dojindo), according to the manufacturer’s instructions. The concentration required to inhibit cell proliferation by 50% (IC_50_) was calculated from the survival curve.

### Cell proliferation assay and colony formation assay

For the cell proliferation assay, 2 × 10^4^ cells were seeded into 12-well plate in triplicate and counted with a Vi-cell analyser (Beckman Coulter). A colony formation assay was performed in triplicate with 1 × 10^4^ cells suspended in 2 ml medium containing 0.35% agarose spread on top of 2 ml medium containing 0.55% agarose. At 14 days after seeding, colonies were stained with crystal violet and counted.

### In silico analysis using ONCOMINE and TCGA datasets

The expression profiles of *PRMT5*, *MEP50*, and GLI target genes in small cell lung carcinoma and skin basal cell carcinoma were obtained from the ONCOMINE database (https://www.oncomine.org/) in May 2018. Datasets used in this study were obtained from the NCBI Gene Expression Omnibus (https://www.ncbi.nlm.nih.gov/geo/ [Small cell lung carcinoma dataset: GSE3398; Gastric cancer dataset: GSE13911; Skin basal cell carcinoma dataset: GSE7553]). The expression profiles of *PRMT5*, *MEP50*, and GLI target genes in stomach and breast cancers were obtained from the UCSC Xena browser (https://xena.ucsc.edu) in August 2018. TCGA stomach adenocarcinoma (STAD) gene expression and TCGA breast invasive carcinoma (BRCA) gene expression in RNAseq (polyA+IlluminaHiSeq UNC) datasets were measured experimentally using the Illumina HiSeq 2000 RNA Sequencing platform by the University of North Carolina TCGA Genome Characterisation Centre. Level 3 data were downloaded from the TCGA Data Coordination Centre.

### Statistical analysis

Statistical testing was performed using the unpaired two-tailed Student’s *t*-test. A value of *P* < 0.05 was considered as statistically significant. Microsoft Excel 2016 was used for statistical analysis. *N* numbers are indicated in the figure legends.

## Supplementary Information

Supplementary Information

Description of Additional Supplementary Files

Supplementary Data 1

Supplementary Data 2

## Data Availability

All data generated or analysed during this study are included in this published Article and its Supplementary files. All full immunoblots are shown in Supplementary Figures 6 and 7. Proteomic mass spectrometry data are available at PeptideAtlas via accession number PASS01311.

## References

[1] Kinzler KW (1987). Identification of an amplified, highly expressed gene in a human glioma. Science.

[2] Ng JM, Curran T (2011). The Hedgehog’s tale: developing strategies for targeting cancer. Nat. Rev. Cancer.

[3] Hui CC, Angers S (2011). Gli proteins in development and disease. Annu. Rev. Cell Dev. Biol..

[4] Ingham PW, McMahon AP (2001). Hedgehog signaling in animal development: paradigms and principles. Genes Dev..

[5] Beachy PA, Karhadkar SS, Berman DM (2004). Tissue repair and stem cell renewal in carcinogenesis. Nature.

[6] Ingham PW, Placzek M (2006). Orchestrating ontogenesis: variations on a theme by sonic hedgehog. Nat. Rev. Genet..

[7] Briscoe J, Therond PP (2013). The mechanisms of Hedgehog signalling and its roles in development and disease. Nat. Rev. Mol. Cell Biol..

[8] Regl G (2002). Human GLI2 and GLI1 are part of a positive feedback mechanism in basal cell carcinoma. Oncogene.

[9] Tukachinsky H, Lopez LV, Salic A (2010). A mechanism for vertebrate Hedgehog signaling: recruitment to cilia and dissociation of SuFu-Gli protein complexes. J. Cell Biol..

[10] Pan Y, Wang B (2007). A novel protein-processing domain in Gli2 and Gli3 differentially blocks complete protein degradation by the proteasome. J. Biol. Chem..

[11] Chen MH (2009). Cilium-independent regulation of Gli protein function by Sufu in Hedgehog signaling is evolutionarily conserved. Genes Dev..

[12] Zhang Q (2009). Multiple Ser/Thr-rich degrons mediate the degradation of Ci/Gli by the Cul3-HIB/SPOP E3 ubiquitin ligase. Proc. Natl Acad. Sci. USA.

[13] Di Marcotullio L (2006). Numb is a suppressor of Hedgehog signalling and targets Gli1 for Itch-dependent ubiquitination. Nat. Cell Biol..

[14] Berman DM (2003). Widespread requirement for Hedgehog ligand stimulation in growth of digestive tract tumours. Nature.

[15] Karhadkar SS (2004). Hedgehog signalling in prostate regeneration, neoplasia and metastasis. Nature.

[16] Thayer SP (2003). Hedgehog is an early and late mediator of pancreatic cancer tumorigenesis. Nature.

[17] Watkins DN (2003). Hedgehog signalling within airway epithelial progenitors and in small-cell lung cancer. Nature.

[18] Theunissen JW, de Sauvage FJ (2009). Paracrine Hedgehog signaling in cancer. Cancer Res..

[19] Merchant AA, Matsui W (2011). Targeting Hedgehog—a cancer stem cell pathway. Clin. Cancer Res..

[20] Mastrangelo E, Milani M (2018). Role and inhibition of GLI1 protein in cancer. Lung Cancer.

[21] Abe Y (2008). Hedgehog signaling overrides p53-mediated tumor suppression by activating Mdm2. Proc. Natl Acad. Sci. USA.

[22] Friesen WJ (2002). A novel WD repeat protein component of the methylosome binds Sm proteins. J. Biol. Chem..

[23] Meister G (2001). Methylation of Sm proteins by a complex containing PRMT5 and the putative U snRNP assembly factor pICln. Curr. Biol..

[24] Gao S, Wu H, Wang F, Wang Z (2010). Altered differentiation and proliferation of prostate epithelium in mice lacking the androgen receptor cofactor p44/WDR77. Endocrinology.

[25] Hosohata K (2003). Purification and identification of a novel complex which is involved in androgen receptor-dependent transcription. Mol. Cell Biol..

[26] Smith TF, Gaitatzes C, Saxena K, Neer EJ (1999). The WD repeat: a common architecture for diverse functions. Trends Biochem. Sci..

[27] Xu C, Min J (2011). Structure and function of WD40 domain proteins. Protein Cell.

[28] Chen JK, Taipale J, Young KE, Maiti T, Beachy PA (2002). Small molecule modulation of smoothened activity. Proc. Natl Acad. Sci. USA.

[29] Vagin VV (2009). Proteomic analysis of murine Piwi proteins reveals a role for arginine methylation in specifying interaction with Tudor family members. Genes Dev..

[30] Tee WW (2010). Prmt5 is essential for early mouse development and acts in the cytoplasm to maintain ES cell pluripotency. Genes Dev..

[31] Karkhanis V, Hu YJ, Baiocchi RA, Imbalzano AN, Sif S (2011). Versatility of PRMT5-induced methylation in growth control and development. Trends Biochem. Sci..

[32] Chiang K (2017). PRMT5 is a critical regulator of breast cancer stem cell function via histone methylation and FOXP1 expression. Cell Rep..

[33] Katoh Y, Katoh M (2009). Hedgehog target genes: mechanisms of carcinogenesis induced by aberrant hedgehog signaling activation. Curr. Mol. Med..

[34] Stecca B, Ruiz IAA (2010). Context-dependent regulation of the GLI code in cancer by HEDGEHOG and non-HEDGEHOG signals. J. Mol. Cell Biol..

[35] Creyghton MP (2010). Histone H3K27ac separates active from poised enhancers and predicts developmental state. Proc. Natl Acad. Sci. USA.

[36] Wang Y (2016). Oncogenic functions of Gli1 in pancreatic adenocarcinoma are supported by its PRMT1-mediated methylation. Cancer Res.

[37] Skrzypczak, M. et al. Modeling oncogenic signaling in colon tumors by multidirectional analyses of microarray data directed for maximization of analytical reliability. *PLoS One* **5**, 10.1371/journal.pone.0013091 (2010). 10.1371/journal.pone.0013091 PMC2948500 20957034

[38] Aberger F, Ruiz IAA (2014). Context-dependent signal integration by the GLI code: the oncogenic load, pathways, modifiers and implications for cancer therapy. Semin. Cell Dev. Biol..

[39] Ding H (2012). Sonic hedgehog signaling mediates epithelial-mesenchymal communication and promotes renal fibrosis. J. Am. Soc. Nephrol..

[40] Sheng T, Chi S, Zhang X, Xie J (2006). Regulation of Gli1 localization by the cAMP/protein kinase A signaling axis through a site near the nuclear localization signal. J. Biol. Chem..

[41] Shi X, Zhan X, Wu J (2015). A positive feedback loop between Gli1 and tyrosine kinase Hck amplifies shh signaling activities in medulloblastoma. Oncogenesis.

[42] Agarwal NK (2016). Active IKKbeta promotes the stability of GLI1 oncogene in diffuse large B-cell lymphoma. Blood.

[43] Coni S, Di Magno L, Canettieri G (2015). Determination of acetylation of the Gli transcription factors. Methods Mol. Biol..

[44] Yang Y, Bedford MT (2013). Protein arginine methyltransferases and cancer. Nat. Rev. Cancer.

[45] Pal S, Vishwanath SN, Erdjument-Bromage H, Tempst P, Sif S (2004). Human SWI/SNF-associated PRMT5 methylates histone H3 arginine 8 and negatively regulates expression of ST7 and NM23 tumor suppressor genes. Mol. Cell Biol..

[46] Antonysamy S (2017). The structure and function of the PRMT5:MEP50 complex. Subcell. Biochem..

[47] Gu Z (2013). The p44/wdr77-dependent cellular proliferation process during lung development is reactivated in lung cancer. Oncogene.

[48] Lingel A (2017). Structure-guided design of EED binders allosterically inhibiting the epigenetic polycomb repressive complex 2 (PRC2) methyltransferase. J. Med. Chem..

[49] Chen H, Lorton B, Gupta V, Shechter D (2017). A TGFbeta-PRMT5-MEP50 axis regulates cancer cell invasion through histone H3 and H4 arginine methylation coupled transcriptional activation and repression. Oncogene.

[50] Didiasova, M., Schaefer, L. & Wygrecka, M. Targeting GLI transcription factors in cancer. *Molecules* **23**, 1003 (2018). 10.3390/molecules23051003 PMC6100584 29695137

[51] Beauchamp EM (2011). Arsenic trioxide inhibits human cancer cell growth and tumor development in mice by blocking Hedgehog/GLI pathway. J. Clin. Invest..

[52] Kayser S, Schlenk RF, Platzbecker U (2018). Management of patients with acute promyelocytic leukemia. Leukemia.

[53] Chan-Penebre E (2015). A selective inhibitor of PRMT5 with in vivo and in vitro potency in MCL models. Nat. Chem. Biol..

[54] Kawauchi K, Araki K, Tobiume K, Tanaka N (2008). p53 regulates glucose metabolism through an IKK-NF-kappaB pathway and inhibits cell transformation. Nat. Cell Biol..

[55] Chang IF (2006). Mass spectrometry-based proteomic analysis of the epitope-tag affinity purified protein complexes in eukaryotes. Proteomics.

