# Supplementary material for: MEP50/PRMT5-mediated methylation activates GLI1 in Hedgehog signalling through inhibition of ubiquitination by the ITCH/NUMB complex
Source: Commun Biol. 2019 Jan 18;2:23. doi: 10.1038/s42003-018-0275-4 (PMC6338668; doi:10.1038/s42003-018-0275-4)
Supplement: Supplementary file 1 — Supplementary Information [file 42003_2018_275_MOESM1_ESM.pdf]

Supplementary Information

MEP50/PRMT5-mediated methylation activates GLI1 in Hedgehog signalling through inhibition of ubiquitination by the ITCH/NUMB complex

Yoshinori Abe, Yosuke Suzuki, Kenji Kawamura and Nobuyuki Tanaka

Supplementary Figure

Supplementary Figure 1

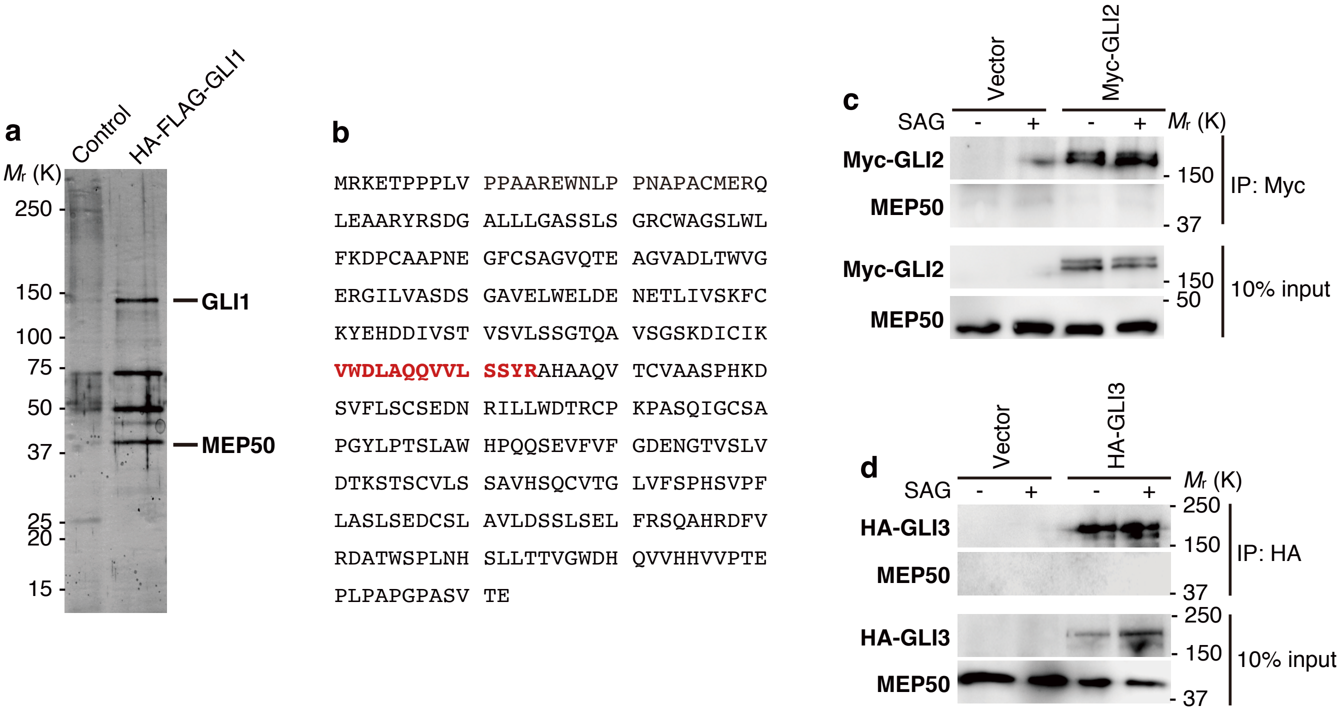

Supplementary Figure 1. MEP50 exclusively interacts with GLI1 and supports GLI1 activation.

(**a**) Silver staining of affinity-purified GLI1 complexes from cytoplasmic extracts of HEK293 cells stably expressing HA-FLAG-GLI1 and control parental HEK293 cells. Specific GLI1-interacting protein bands were analyzed by mass spectrometry. (**b**) Sequence of MEP50. Peptides (indicated in red) eluted from the tryptic digest of a 45 kDa polypeptide co-purified with HA-FLAG-GLI1 matched the sequence of human MEP50. (**c** and **d**) Interactions between Myc-GLI2 (c) or HA-GLI3 (d) and endogenous MEP50 in C3H10T1/2 cells. At 24 h post-transfection, cells were treated with 300 nM SAG for 24 h and then cells were lysed and subjected to immunoprecipitation with an anti-Myc (c) or anti-HA (d) followed by immunoblot with an anti-MEP50 (3F10) antibody. Unprocessed original scans of blots are shown in Supplementary Fig. 7.

Supplementary Figure 2

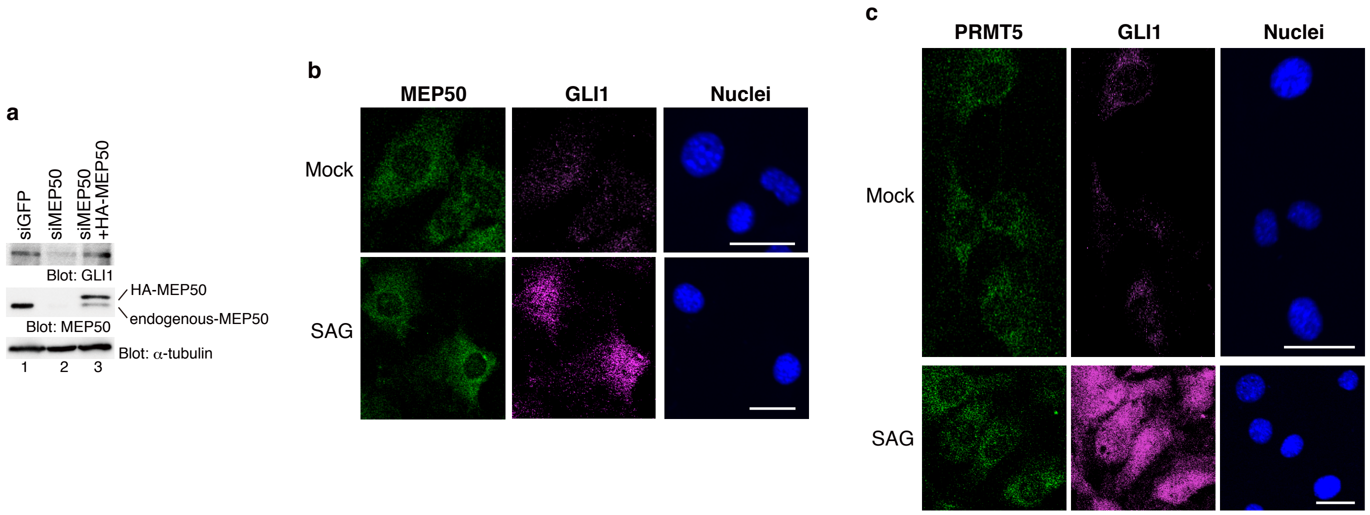

Supplementary Figure 2. The MEP50/PRMT5 complex supports GLI1 activation through GLI1 stabilization downstream of SMO.

(a) Immunoblot analysis of Gli1 and MEP50 in C3H10T1/2 cells in which MEP50 was knocked down by siMEP50-m2 (lane 2) and then rescued with a siRNA-resistant HA-human MEP50 (lane 3). siRNA was stably expressed by a recombinant retrovirus. Proteins were detected with the indicated antibodies. (**b** and **c**) Subcellular localization of MEP50 (b) or PRMT5 (c) and GLI1 in C3H10T1/2 cells. SAG (300 nM) was applied for 36 h. Scale bars: 40 μm. Unprocessed original scans of blots are shown in Supplementary Fig. 7.

Supplementary Figure 3

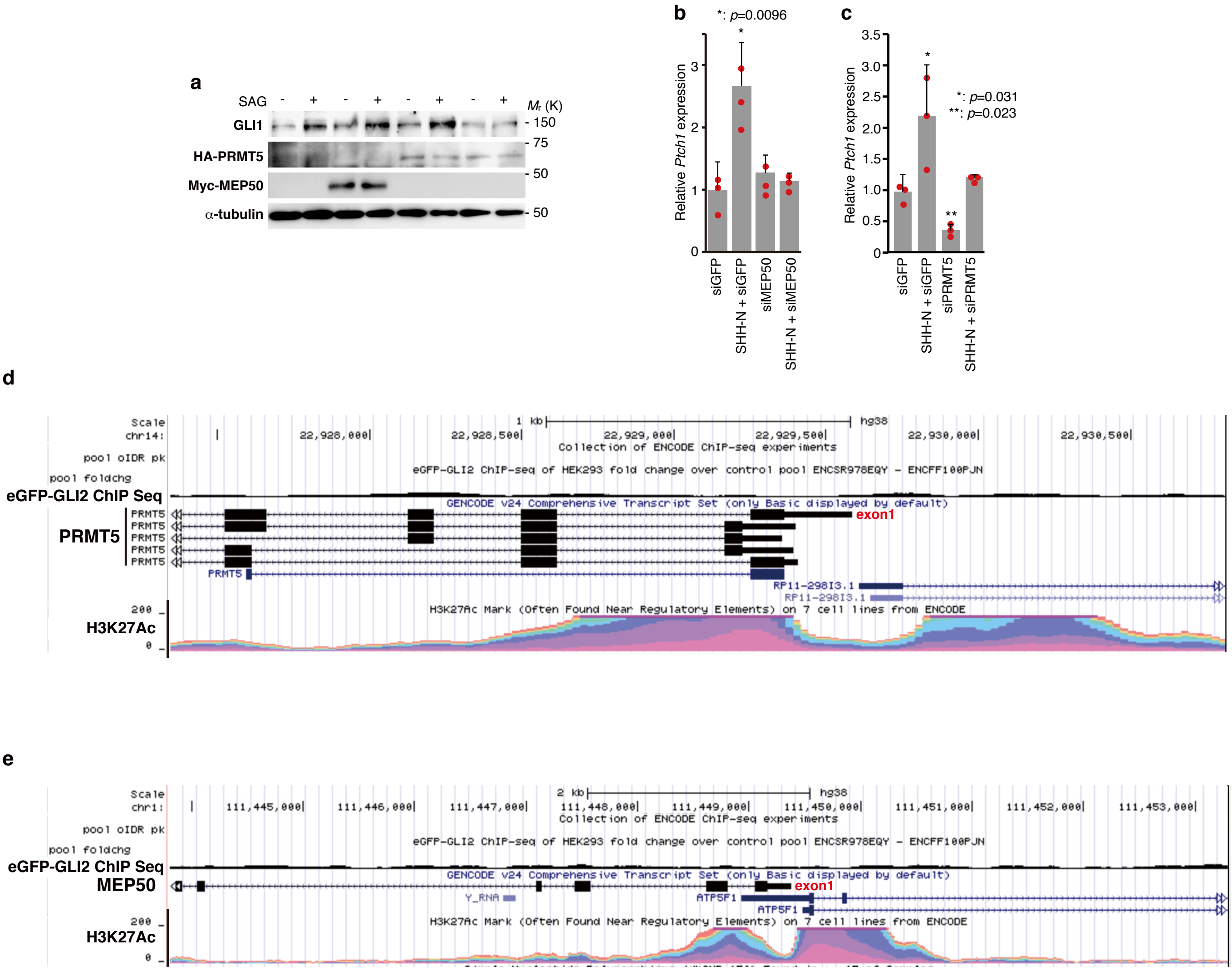

Supplementary Figure 3. GLI2 potentially regulates PRMT5 and MEP50 expression.

(**a**) Immunoblot analysis of GLI1, HA-PRMT5, HA-PRMT5 G367A/R368A, and Myc-MEP50 protein levels in Fig. 3d. (**b** and **c**) Quantitative real-time PCR (qRT-PCR) analysis of *Ptch1* expression in C3H10T1/2 cells knocked down for MEP50 (**b**) or PRMT5 (**c**) and treated with recombinant active SHH (SHH-N; 3 μg/ml) for 24 h as indicated. (**d** and **e**) The UCSC Genome Browser (http://genome.ucsc.edu/index.html) result showing the locations of GLI2 ChIP-Seq, and acetylated histone H3 at Lys27 (H3K27Ac) ChIP-Seq signals on the *PRMT5* (**d**) or *MEP50* (**e**) locus. GLI2 ChIP-Seq data was obtained from GFP-tagged GLI2 expressing HEK293T cells. H3K27Ac ChIP-Seq data was obtained from 7 cell lines from ENCODE. Unprocessed original scans of blots are shown in Supplementary Fig. 7.

Supplementary Figure 4

GLI1-410-600

Homo sapiens	511	PIGT**R**GLKLP	520	591	ARYASA**R**GGG	600
Mus musculus	513	PIGS**R**GLKLP	522	593	ARYASA**R**GSG	602
Rattus norvegicus	510	PIGS**R**GLKLP	519	590	ARYASA**R**GSG	599
Macaca mulatta	511	PIGT**R**GLKLP	520	591	ARYASV**R**GGG	600
Bos taurus	511	PMGP**R**GLKLP	520	591	ARYASA**R**GGG	600
Canis lupus familiaris	510	PIGP**R**GLKLP	519	590	ARYASA**R**GGG	599

GLI1-851-1106

Homo sapiens	871	DYLPSEP**R**PC	880	911	EGGG**R**EDAPA	920	981	ASH**R**AAAPP**R**	990	1011	CGHPEVG**R**LG	1020
Mus musculus	872	GYLSTEP**R**LG	881	912	EGRN**R**GGLPN	921	986	PSH**R**PAAPP**R**	995	1016	CGHPEVG**R**LG	1025
Rattus norvegicus	869	DCLSLES**R**PG	878	909	EGRS**R**GGIPN	918	984	PPH**R**PAAPP**R**	993	1014	CGHPEVG**R**LG	1023
Macaca mulatta	871	DYLPSEP**R**PC	880	911	EGGG**R**EDASA	920	981	ASH**R**AAAPP**R**	990	1011	CGHPEVG**R**LG	1020
Bos taurus	871	DYLPSEA**R**PS	880	911	DSGG**R**GDPPV	920	980	ASH**R**AAAPP**R**	989	1010	CGHPEVG**R**LG	1019
Canis lupus familiaris	870	DYLPSEP**R**PA	879	910	DGGG**R**GDPPV	919	980	TSH**R**AAAPP**R**	989	1010	CGHPEVG**R**LG	1019

Supplementary Figure 4. Conserved arginine residues methylated by PRMT5 in GLI1.

Seven arginine sites (positions 515, 597, 878, 915, 984, 990 and 1018) are arginine residues that are candidate methylation sites in PRMT5. These seven sites are conserved in six mammalian species.

Supplementary Figure 5

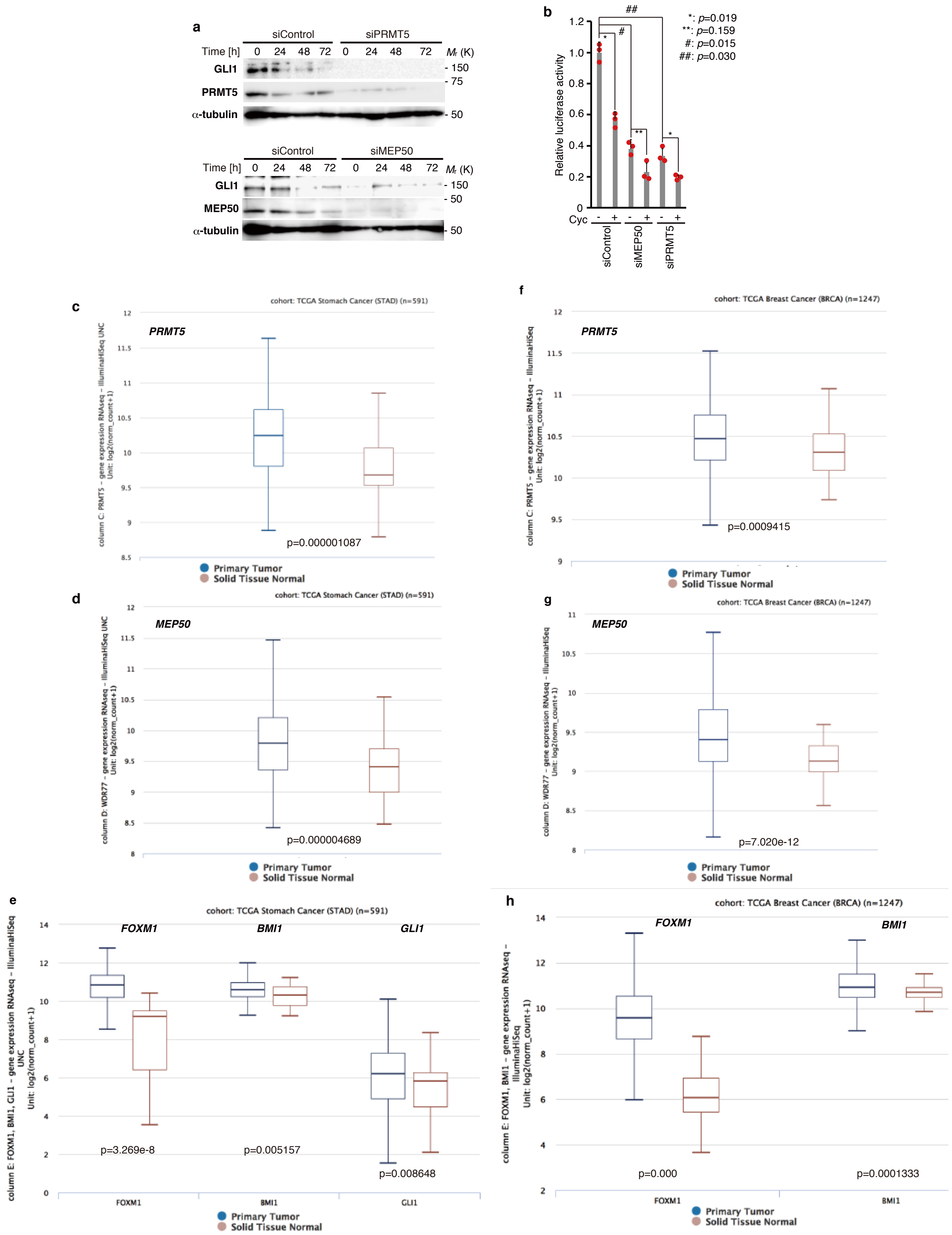

Supplementary Figure 5. Suppression of PRMT5 or MEP50 sensitizes to a SMO inhibitor, and PRMT5, MEP50, and GLI1 target genes are upregulated in stomach and breast cancers.

(a) Immunoblot analysis of GLI1 protein levels in stable siControl-, siMEP50-, or siPRMT5 siRNA-expressing cells treated with cyclopamine (final concentration: 10 μM) for the indicated times. siRNAs were stably expressed by recombinant retroviruses. **(b)** Gli transcriptional activity in stable MEP50 or PRMT5 knockdown AGS cells. siRNAs were stably expressed by recombinant retroviruses. A multimerized Gli-binding site luciferase reporter plasmid and phRL-TK control reporter plasmid were transfected into cells. After 24 h of incubation, 10 μM cyclopamine (Cyc) was applied for 48 h, and then luciferase assays were performed. Results are shown as the mean ± s.d. of triplicates. Data represent one of two independent experiments with similar results. (**c–e**) Upregulated expression of *PRMT5*, *MEP50*, and GLI1 target genes in stomach cancer. (**f–h**) Upregulated expression of *PRMT5*, *MEP50*, and GLI1 target genes in breast cancer. Datasets were obtained from the UCSC Xena browser (https://xena.ucsc.edu) of The Cancer Genome Atlas (TCGA). The threshold of data was $p < 0.05$. Each boxplot shows the $\log_2$ maximum, minimum, and median signal intensity of each mRNA from the corresponding expression array. Bold lines on each boxplot define the median value. Unprocessed original scans of blots are shown in Supplementary Fig. 7.

Supplementary Figure 6

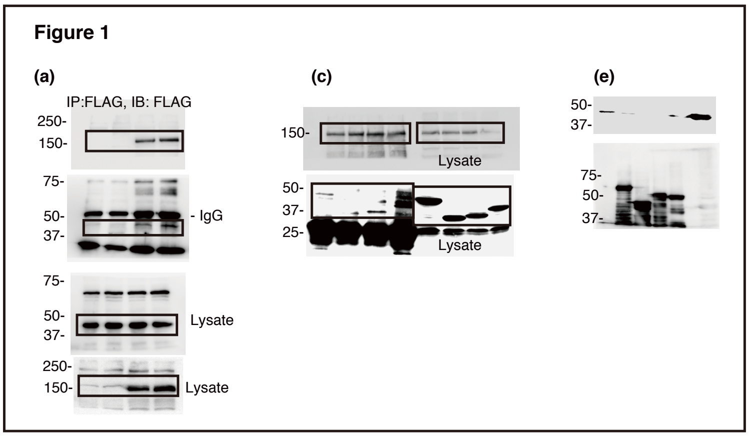

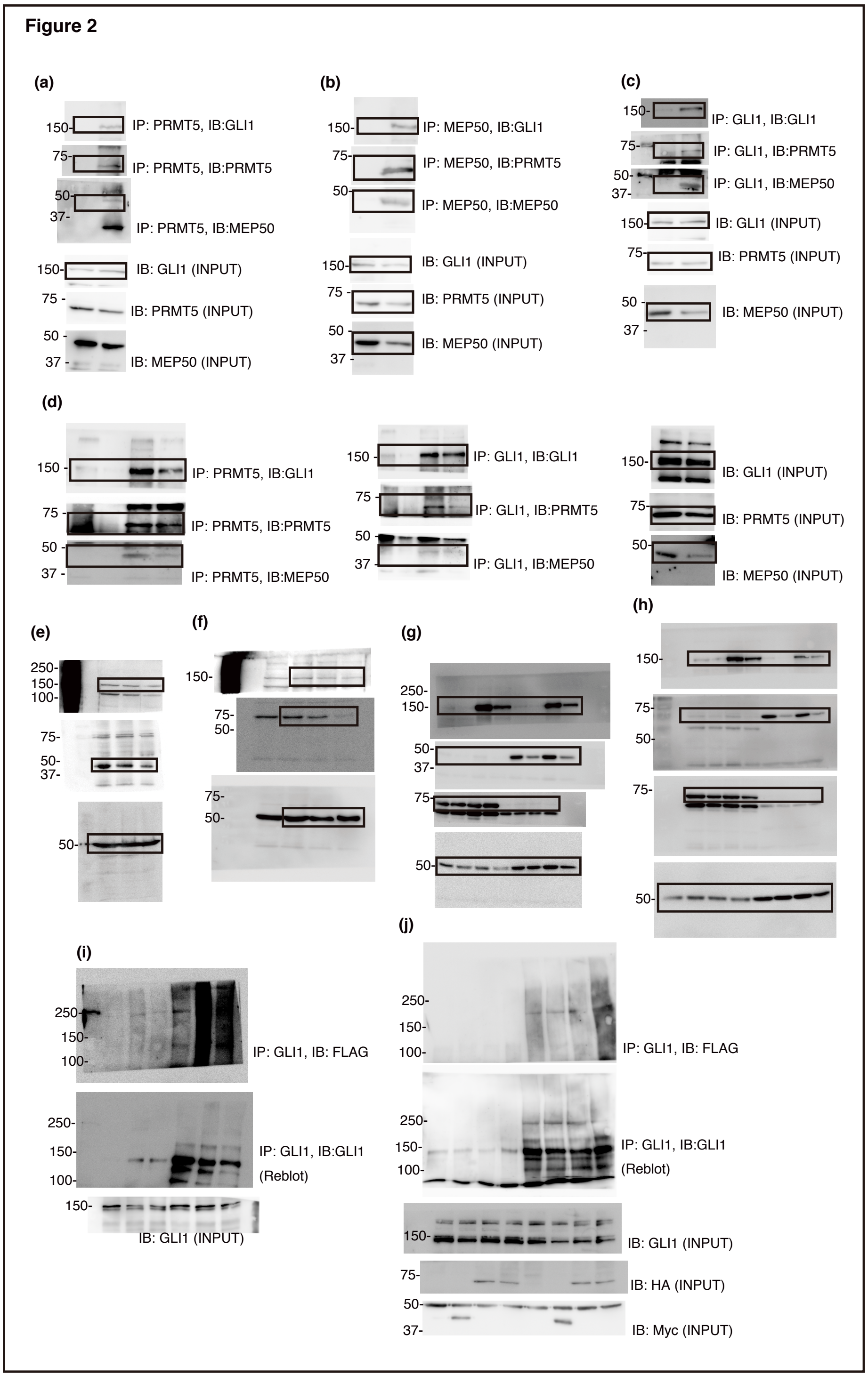

Supplementary Figure 6 (continued)

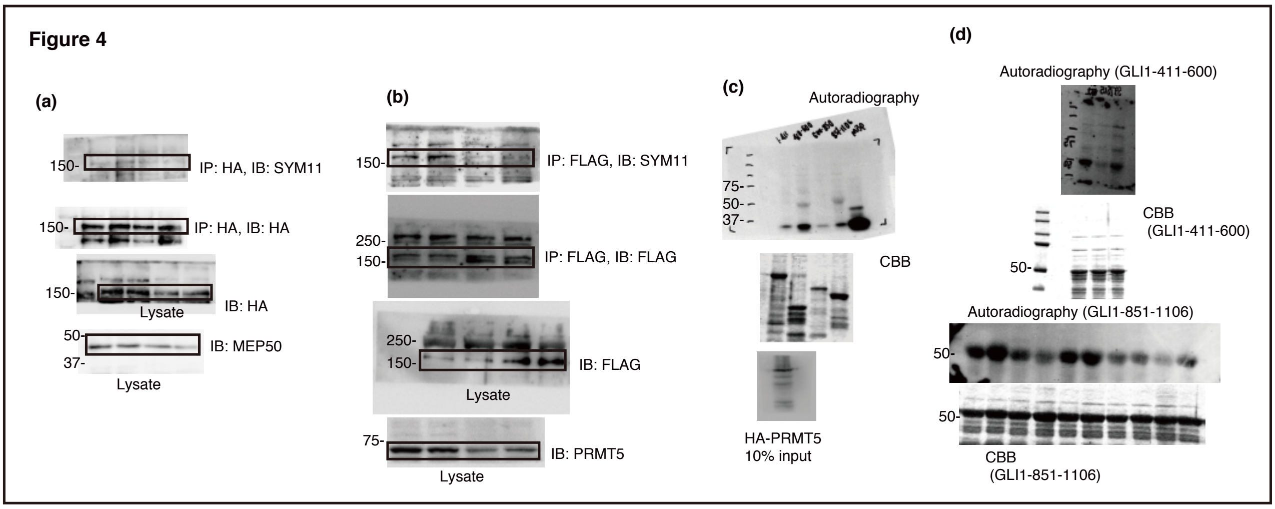

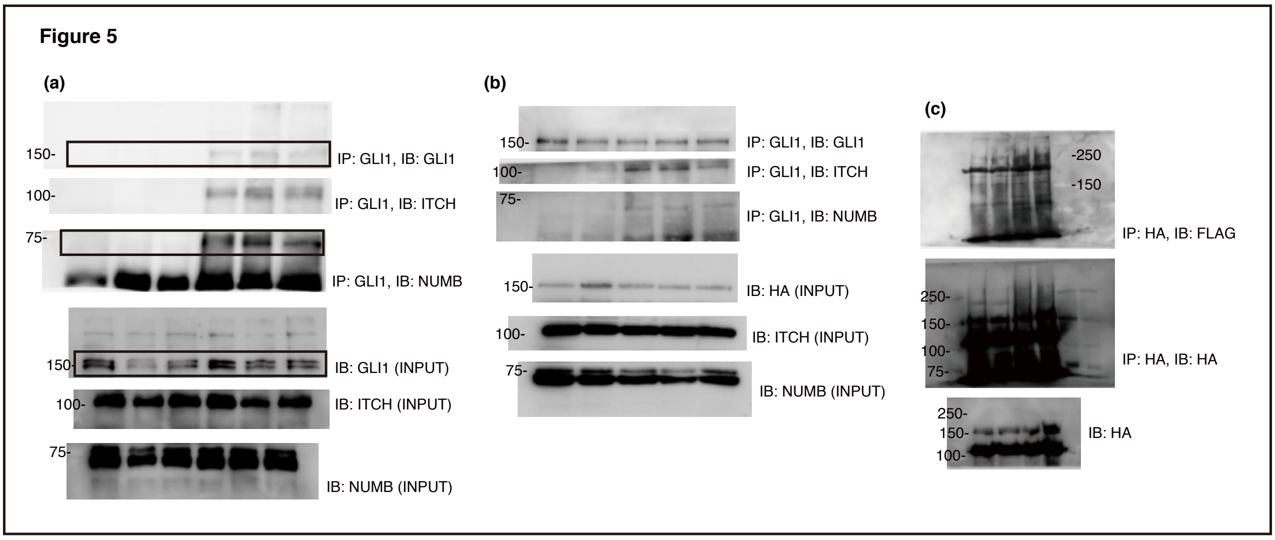

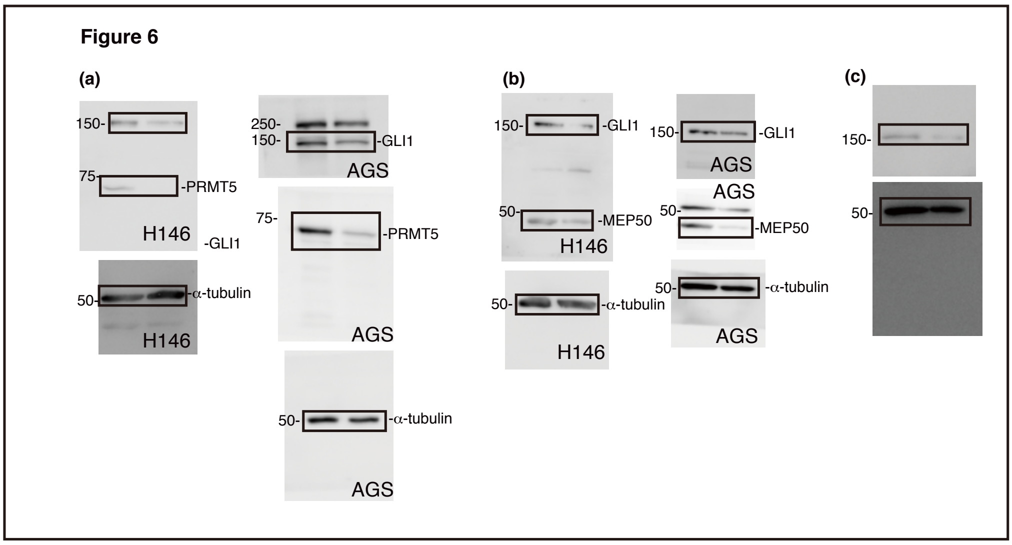

Supplementary Figure 6. Unprocessed original scans of blots represented in Figures 1–6.

Supplementary Figure 7

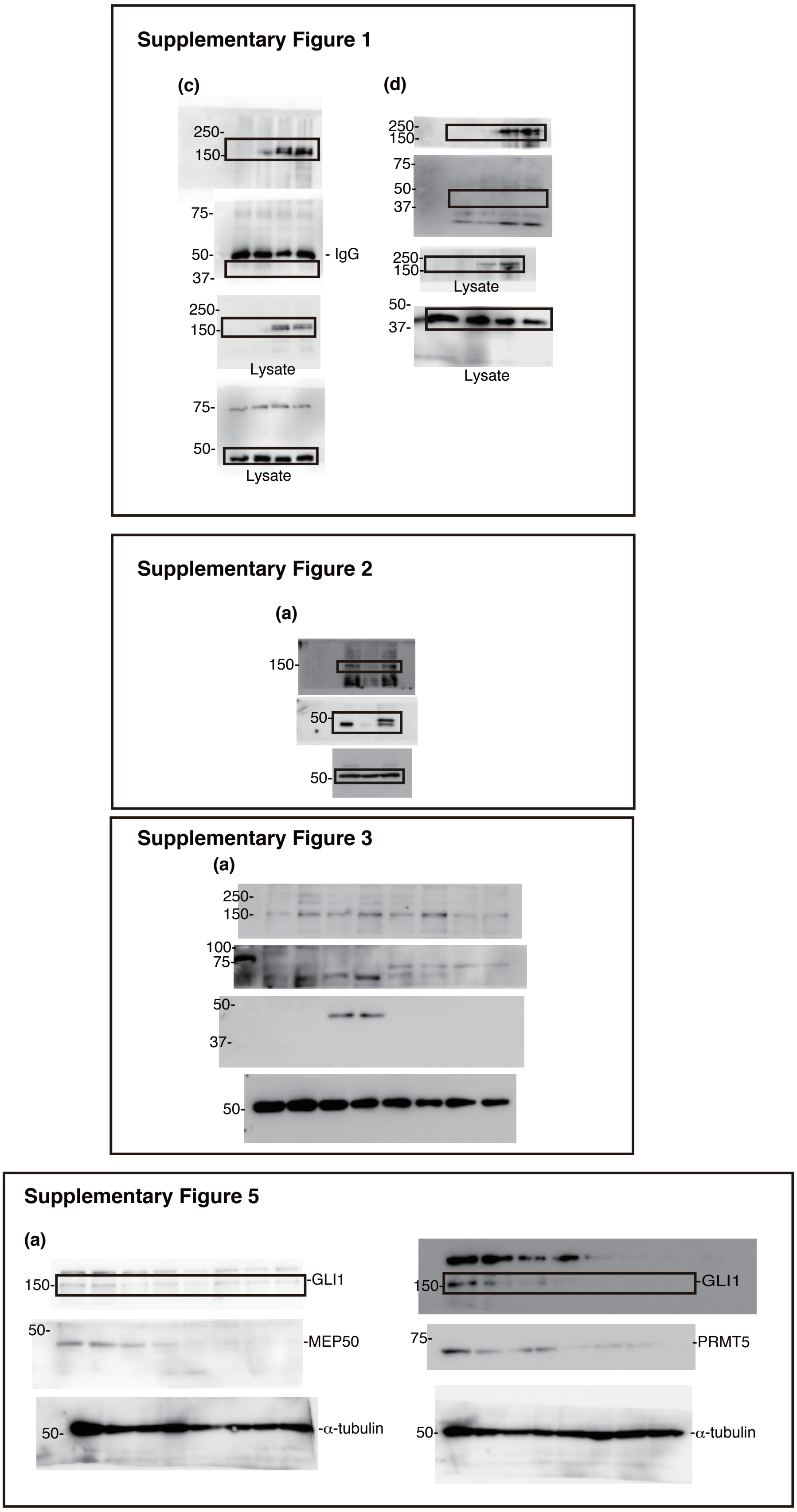

Supplementary Figure 7. Unprocessed original scans of blots represented in Supplementary Figures 1–5.
