# Supplementary material for: MEP50/PRMT5-mediated methylation activates GLI1 in Hedgehog signalling through inhibition of ubiquitination by the ITCH/NUMB complex
Source: Commun Biol. 2019 Jan 18;2:23. doi: 10.1038/s42003-018-0275-4 (PMC6338668; doi:10.1038/s42003-018-0275-4)
Supplement: Supplementary file 2 — Description of Additional Supplementary Files [file 42003_2018_275_MOESM2_ESM.pdf]

Description of Additional Supplementary Files

File name: Supplementary_Data_1
Description: Source data of Figure 3.

File name: Supplementary_Data_2
Description: Source data of Figure 6d–f.
